# T‐cell responses against rhinovirus species A and C in asthmatic and healthy children

**DOI:** 10.1002/iid3.206

**Published:** 2017-11-10

**Authors:** Cibele M. Gaido, Caitlyn Granland, Ingrid A. Laing, Peter N.Le Souëf, Wayne R. Thomas, Andrew J. Currie, Belinda J. Hales

**Affiliations:** ^1^ Telethon Kids Institute The University of Western Australia Perth Australia; ^2^ School of Paediatrics and Child Health The University of Western Australia Perth Australia; ^3^ Princess Margaret Hospital for Children Perth Australia; ^4^ School of Veterinary & Life Sciences Murdoch University Perth Australia

**Keywords:** Asthma, regulatory T‐cells, rhinovirus, T‐cell proliferation

## Abstract

**Background:**

Infections by rhinovirus (RV) species A and C are the most common causes of exacerbations of asthma and a major cause of exacerbations of other acute and chronic respiratory diseases. Infections by both species are prevalent in pre‐school and school‐aged children and, particularly for RV‐C, can cause severe symptoms and a need for hospitalization. While associations between RV infection and asthma are well established, the adaptive immune‐mechanisms by which RV infections influence asthma exacerbations are yet to be defined.

**Objective:**

The aim of this study was to characterize and compare T‐cell responses between RV‐A and RV‐C and to test the hypothesis that T‐cell responses would differ between asthmatic children and healthy controls.

**Methods:**

A multi‐parameter flow cytometry assay was used to characterize the in vitro recall T‐cell response against RV‐A and RV‐C in PBMCs from children with acute asthma (*n* = 22) and controls (*n* = 26). The responses were induced by pools of peptides containing species‐specific VP1 epitopes of RV‐A and RV‐C.

**Results:**

Regardless of children's clinical status, all children that responded to the in vitro stimulation (>90%) had a similar magnitude of CD4+ T‐cell responses to RV‐A and RV‐C. However, asthmatic children had a significantly lower number of circulating regulatory T cells (Tregs), and healthy controls had significantly more Tregs induced by RV‐A than RV‐C.

**Conclusions and Clinical Relevance:**

The comparable recall memory T‐cell responses in asthmatic and control children to both RV‐A and RV‐C show that differences in the antibody and inflammatory responses previously described are likely to be due to regulation, with a demonstrated candidate being reduced regulatory T‐cells. The reduced Treg numbers demonstrated here could explain the asthmatic's inability to appropriately control immunopathological responses to RV infections.

## Introduction

Rhinovirus (RV) infections are the major cause of acute asthma exacerbations 1, 2 and are also important causes of acute lower respiratory infections in cystic fibrosis 3 and chronic obstructive pulmonary disease 4. School‐aged children are likely to have recurrent RV infections 5, 6, thus are at particular risk for RV‐induced asthma exacerbations. Host genetics in combination with environmental factors (i.e., atopy) and characteristics of the virus are known to influence the severity of RV‐induced asthma 7, 8. From the three RV species currently identified, RV‐A and RV‐C are the most prevalent amongst children 9, 10, 11 and RV‐C has been associated with more severe symptoms of infection and a higher frequency of asthma exacerbations 12, 13. While the association between RV infections and asthma exacerbations are now well established, the mechanisms by which RV infections induce asthma exacerbations are yet to be defined.

A recent study utilizing peptide/MHC class II tetramer‐guided epitope mapping has identified circulating epitope‐specific memory CD4+ cells to a genotype of RV‐A, indicating that recall memory CD4+ T cell responses drive the immune response against RV infections. Furthermore, intra‐species cross‐reactivity was evidenced by in vitro proliferation of RV‐A16‐induced‐memory T cells to peptides from another RV‐A genotype, RV‐A39 14, indicating that the memory response to RV is less type‐specific than what was previously expected. This study was conducted in healthy adult donors, therefore the adaptive response to RV in asthmatic children remains unknown. The only study on adaptive responses of asthmatic children to RV‐C focused on antibody responses and reported markedly lower and often undetectable species specific IgG1 antibody titres to their VP1 proteins, contrasting to the high specific titres to the VP1 proteins of RV‐A 15. Furthermore, the anti‐RV‐A antibody responses but not those specific to RV‐C were higher in asthmatics compared with controls. These unexpected and intriguing differences might be due to overall poor immune response to RV‐C that could be evident in the T‐cell compartment which is examined here.

The current study describes T‐cell responses to RV‐A and RV‐C in a pediatric clinical cohort of asthma‐diagnosed children. The responses measured are induced by pools of peptides containing epitopes of the RV‐A and RV‐C VP1 proteins that are both species‐specific and representative of memory T‐cell responses made to each species 16. We aimed to characterize and compare T‐cell responses between RV‐A and RV‐C and to test the hypothesis that T‐cell responses would differ between asthmatic children and healthy controls.

## Methods

### Study participants

Biobanked peripheral blood mononuclear cells (PBMCs) and plasma of 48 children (aged 5 months to 14 years) were collected between December 2009 and March 2015 as part of the “Mechanisms of Viral Infection in Children” (MAVRIC) cohort, from Princess Margaret Hospital for Children (Perth, Western Australia). The MAVRIC study is a continuation of the Perth childhood acute asthma study began by Professor Peter Le Souëf where the role of virus infection in childhood respiratory illness is being investigated in a range of genetic and inflammation related studies. Children presenting with an acute wheezing episode to the emergency department (ED) were recruited into the study and examined by an independent physician for asthma and wheeze assessments and nasal samples were taken and submitted to routine virus diagnosis and testing for RV species. Blood sample have been taken at admission and during the follow‐up which is part of the study. The blood samples (5–10 mL), used here were collected after the children had recovered from their acute episode of asthma, were diluted with an equal volume of RPMI‐1640 medium (Sigma–Aldrich, St Louis, USA) and 10 IU/mL preservative‐free heparin (Pfizer, New York, USA). The PBMCs were isolated by density gradient Lymphoprep™ (Nycomed, Oslo, Norway) and washed three times in RPMI media prior to cryopreservation. From the cohort, 22 children (asthmatics, mean age in years 7.7 ± 3.7) had been diagnosed with asthma, requiring hospitalization (Table 1). Children with no medical history of doctor‐diagnosed asthma or other respiratory illness were included as controls (mean age in years 5.9 ± 3.7, *n* = 26). All 48 children (asthmatics and controls) had nasal secretion specimens tested for RV using a molecular detection and typing assay 12 to determine the RV genotypes. As shown in Table 1 and reported previously 1, 15, 17, most asthmatic children presenting to the ED following an epidose of asthma exacerbation had detectable RV (70%) and the majority of the infections were caused by RV‐C. 27% of the nasal swabs, taken at the time of blood collection, from the healthy controls were PCR+ for RV in keeping with other studies of disease free children.

**Table 1:** Characteristics of the study population

Characteristic	^a^Asthmatics, *n* = 22	Controls, *n* = 26
Mean age ± SD (y)	7.7 ± 3.7	5.9 ± 3.7
Age range (y)	2.1–14.3	0.5–14.1
Female, *n* (%)	13 (60)	16 (60)
Atopic, *n* (%)	18 (80)	6 (20)
RV positive, *n* (%)	15 (70)	7 (27)
RV‐A, *n*	3	1
RV‐B, *n*	1	2
RV‐C, *n*	11	4

^a^ Doctor‐diagnosed asthmatic children. Period in months between the last episode of asthma exacerbation, at recruitment, and sample collection: 5.1 months ± 2 months.

PBMCs from asthmatics were collected at their follow‐up visit, 2–9 months (mean 5 months) after an acute episode of asthma resulting in an ED presentation. The choice of using samples collected at the follow‐up visit, when the children were clinically well, was to avoid external confounders (i.e., corticosteroid administration) that could potentially impact the in vitro adaptive immune responses. The research has been approved by the Princess Margaret Hospital Human Ethics Committee (approval number 1761/EP) and written informed consent was obtained from each parental/guardian of all participants.

### Rhinovirus peptides

Immunodominant peptides of the VP1 capsid protein of rhinoviruses A and C, recently identified as specific for and representative of each RV‐A and RV‐C species by an epitope‐mapping study 16, were combined into pools representing each species (Table 2). From a measure that considered the size of the stimulations of individual subjects and the frequency of stimulation in the sample (reactivity score) these peptides accounted for almost 50% of the reactivity score achieved with all the peptides for both RV‐A and RV‐C. The peptides representing each species were in different positions of the VP1 sequence and in regions where there was little response to peptides of the other species and where the sequences between species were 40–85% disparate.

**Table 2:** Pools of RV‐A (Pool A) and RV‐C (Pool C) synthetic peptides of the VP1 capsid protein of RV‐A34 and RV‐C3, respectively

Peptide ID	Sequence^a^
Pool A	
RVA‐23	RKFEMFTYVRFDSEV
RVA‐24	FTYVRFDSEVTLVPS
RVA‐25	FDSEVTLVPSIAAKG
RVA‐48	TTRVYHKAKHVKTWC
RVA‐49	HKAKHVKTWCPRPPR
Pool C	
RVC‐07	PQALGAVEIGATADV
RVC‐16	LWANLRLDQGFRKWE
RVC‐32	PNSGFPRFTIPFTGLG
RVC‐40	LTNDMGTLCFRALDG
RVC‐42	RALDGTGASDIKVFG

^a^ All peptides presented a H‐group at the N‐termini and −OH group at the C‐termini end.

### T‐cell response against RV‐A and RV‐C

Each step of the T‐cell activation process was followed by evaluating three parameters of the CD4+ and CD8+ activation in the in vitro RV response: (i) the co‐expression of the T‐cell activation markers CD25^hi^HLA‐DR^hi^; (ii) the expression of the co‐stimulatory molecule ICOS‐I^hi^; and (iii) effective proliferation, measured by the dilution of the cell‐proliferation dye CellTrace™ Violet into daughter cells.

#### CellTrace™ labeling

Thawed PBMCs were labeled with CellTrace™ Violet proliferation kit (Invitrogen, Mulgrave/USA) as follows: PBMCs were re‐suspended at 6 × 10^6^ cells in 1 mL of PBS in a 15 mL conical tube. The tube was laid horizontally and 110 μL of PBS was added to the non‐wetted portion of the plastic, at the top of the tube. To this, 1.1 μL of CellTrace™ stock solution (5 mM) was re‐suspended to obtain a 5 μM final working concentration. The cell suspension was quickly vortexed to ensure uniform labeling prior to incubation for 5 min at RT, protected from light. Unbound dye was quenched by three consecutive washes with RPMI supplemented with 10% heat inactivated fetal calf serum (HI‐FCS; SAFC, Brooklyn, Australia). At the final washing step, CellTrace™ labeled cells were re‐suspended in warm AIM‐V serum free culture media (Life Technologies, Mulgrave/Australia) supplemented with 50 µM 2‐mercaptoethanol (Invitrogen, Mulgrave/Australia) and seeded at 3 × 10^5^ cells per well in a round bottom 96‐well plate.

#### In vitro stimulation of RV‐A and RV‐C‐specific T cells

PBMCs’ responses measured after a 6‐day stimulation period in the absence of added co‐stimuli (i.e., exogenous cytokines) or enrichment of dendritic cells necessary for in vitro priming of naïve CD4+ cells 18, 19 are memory T‐cell responses. This is also confirmed by the expression of HLA‐DR (see results) known to be expressed by memory and not naïve T cells 20. Seeded wells were cultured in sextuplicate without stimulus (negative control) or stimulated with either RV pools (Table 2) at 15 µM per peptide or the positive control antigen Staphylococcal enterotoxin B (SEB; Sigma‐Aldrich/St Louis/USA) at 2.5 µg/mL for 6 days at 37°C in a 5% CO_2_ atmosphere.

#### Characterization of recall of memory T‐cell response to RV‐A and RV‐C

At day 6, the replicated wells were pooled and cells co‐stained with fluorochrome‐conjugated human specific antibodies against the lineage markers CD3 (APC‐H7, clone SK7), CD4 (FITC, clone RPA‐T4), CD8 (PE‐Cy7, clone RPA‐T8), and the T‐cell activation markers HLA‐DR (PE‐CF594, clone G46‐6), CD25 (PE, clone M‐A251), and ICOS‐I (BV650, clone DX29) in order to determine the activated (CD25^hi^HLA‐DR^hi^ and ICOS‐I^hi^) and proliferating (CellTrace^dim^) cell subset in response to the RV stimulus. CD19/20 (PerCP‐Cy5.5, clones SJ25C1 and L27, respectively) and LIVE/DEAD Viability kit (Invitrogen) were used to exclude B cells and nonviable cells, respectively, while CD25 (PE, clone M‐A251) and CD127 (BV786, clone HIL‐7R‐M21) were used to identify the T‐reg cell subset (all from Becton Dickinson Pharmingen, NJ, USA). Briefly, Prior to staining, cells were washed once to remove culture media. Pooled PBMCs were re‐suspended in PBS and stained with Live/Dead dye according to manufacturer's instruction. After incubation, stained cells were washed twice in PBS containing 1% BSA and 0.1% sodium azide (FACS buffer) to remove excess dye. PBMCs were then stained with the antibody cocktail for 30 min at 4°C. Unbound antibodies were washed out and cells were fixed using Stabilizing Fixative (Becton Dickinson Pharmingen, NJ, USA). Flow cytometry data was acquired using FACS Diva Software on a LSR Fortessa flow cytometer (all from BD Biosciences). At least 250,000 total events were acquired per each stimulation condition. Data was acquired using FlowJo X version 10.0.7r2 (Tree Star). ICOS‐I readings are not available for two cases and two controls due to a shortage of antibody markers from supplier delays.

#### Data analysis

**Figure 1:**
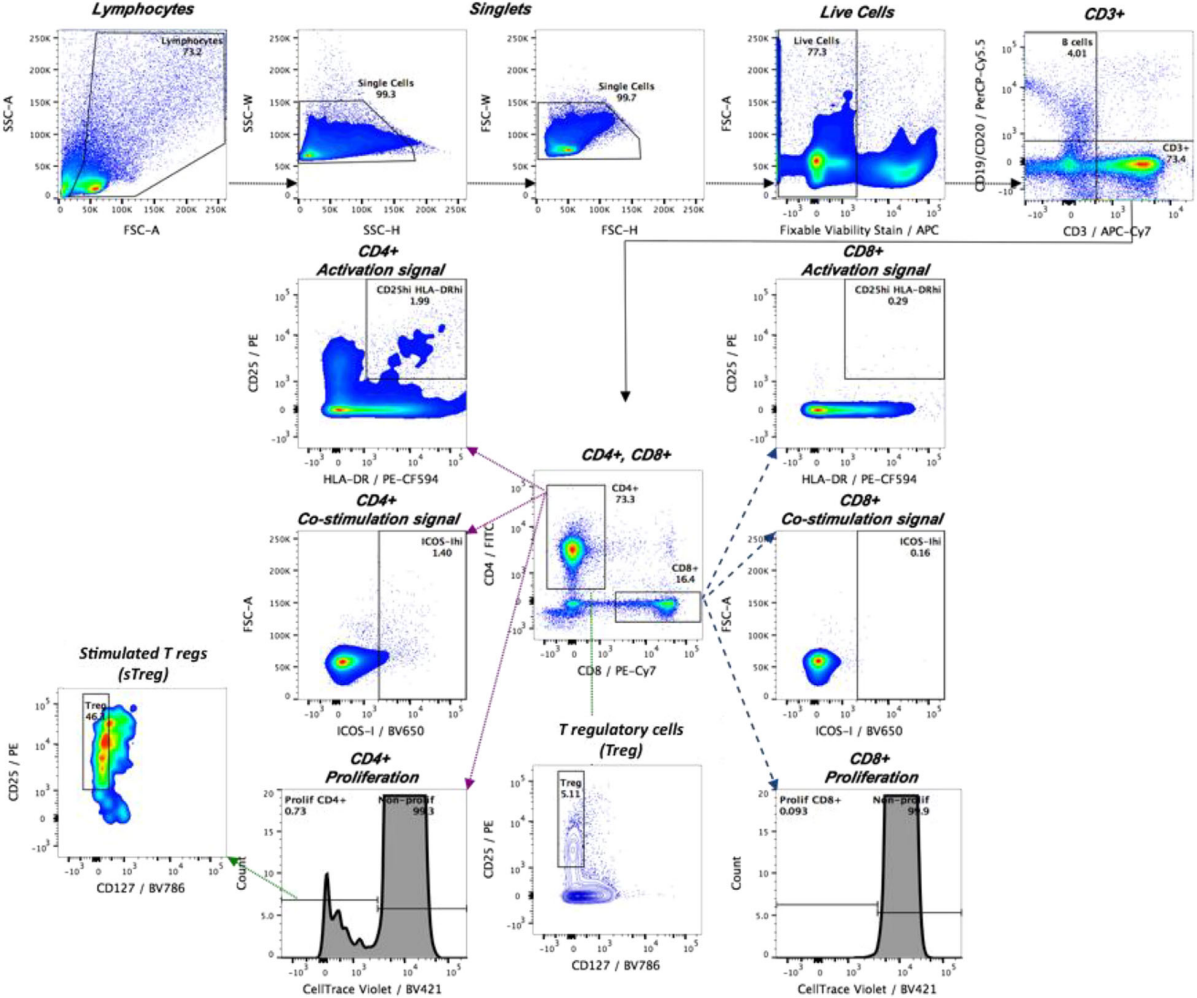
Gating strategy. After exclusion of doublets, dead and B cells, CD4+ and CD8+ were analyzed for T‐cell activation according to the surface expression of the pair of T‐cell activation markers (CD25^hi^HLA‐DR^hi^), the expression of the co‐stimulatory molecule (ICOS‐I^hi^), and the proliferative response (CellTrace^dim^). Tregs (CD4 + CD25^hi^CD127^low^) and sTregs (CD4 + CellTrace^dim^CD25^hi^CD127^low^) were measured in unstimulated and RV‐stimulated cultures, respectively.

Following cell population gating (Fig. 1) on lymphocytes, singlets, live cells, CD3^+^, CD4^+^/CD8^+^, circulating Tregs (CD4 + CD25^hi^CD127^low^) from total CD4+, and stimulated Tregs (sTregs: CD4 + CellTrace^dim^CD25^hi^CD127^low^) from the proliferative CD4+ subset, the percentage of activated (CD25^hi^HLA‐DR^hi^ and ICOS‐I^hi^) and proliferating (CellTrace^dim^) CD4+ and CD8+ cells was recorded for each condition. Activated CD4+ or CD8+ cells were defined as [Δ % of CD25^hi^HLA‐DR^hi^] = (% of CD25^hi^HLA‐DR^hi^ cells among a pool of six antigen‐stimulated wells)−(% of CD25^hi^HLA‐DR^hi^ cells among a pool of six unstimulated control wells) and the same analysis was repeated for ICOS‐I^hi^. The proliferative response was given by the % of CD4+ and CD8+ cell proliferation above background, which was calculated as [Δ % of proliferating cells] = (% of CellTrace^dim^ cells among a pool of six antigen‐stimulated wells)−(% of CellTrace^dim^ cells among a pool of six unstimulated control wells). The threshold for proliferation and activation of 0.01% was used as detection limit of the assay for all antigens for both CD4+ and CD8+ cell subsets. Although there are several cell markers recognized for characterizing different Treg subsets, here we adopted the combination of the cell surface markers CD4 + CD25^hi^CD127^low^ to identify frequency of circulating Tregs in peripheral blood, as described previosuly 21, 22, 23.

### Statistical methods

Non‐parametric RV‐specific CD4+ and CD8+ T cell data was statistically analyzed using independent samples Mann–Whitney U‐tests when comparing between asthmatics and controls, while all paired samples (comparison between RV‐A and RV‐C responses within asthmatics or controls) were compared using related samples Wilcoxon signed rank tests. Chi^2 was used for two sample prevalences comparisons. Differences and associations were considered significant (*) if the *p* value was less than 0.05, while non‐significant results are represented by “ns.” Statistical analyses were performed using the statistical packages Prism (GraphPad Software Inc., La Jolla, USA).

## Results

### Prevalence of the recall T‐cell responses to RV‐A and RV‐Cs in children

The prevalence of CD4+ T‐cell responses to RV‐A and RV‐C was evaluated by investigating the prevalence of antigen specific CD4+ cells expressing the activation and co‐stimulatory markers CD25^hi^HLA‐DR^hi^ and/or ICOS‐I^hi^ and the prevalence of cells progressing to proliferation (CellTrace^dim^). Approximately 70% of the asthmatic children had RV‐A and RV‐C specific CD4+ activation and proliferation, which was similar to the prevalence of RV‐A and RV‐C specific CD4+ activation and proliferation found in control children (Table 3). Proliferation and activation for the CD8+ cells was also found but it was numerically less prevalent than the CD4+ cells, being about 75% for activation and about 50% for the proliferation to both the RV‐A and RV‐C peptides, although not statistically significantly different from the corresponding CD4+ responses (chi2 > *p* = 0.1) (Table 3).

**Table 3:** Prevalence of in vitro T‐cell response to RV‐A and RV‐C, given by the prevalence of RV‐activated and proliferating T cells in asthmatic and control children

	RV‐A		RV‐C	
Study population	Activated	Proliferating	Activated	Proliferating
CD4+				
Asthmatics, *n* (%)	18 (82)	19 (86)	17 (77)	15 (68)
Controls, *n* (%)	20 (80)	19 (75)	18 (70)	18 (70)
CD8+				
Asthmatics, *n* (%)	17 (77)	16 (73)	17 (77)	11 (50)
Controls, *n* (%)	17 (65)	16 (60)	19 (75)	13 (50)

Activated, CD25^hi^HLA‐DR^hi^ and/or ICOS‐I^hi^; Proliferating, CellTrace^dim^.

### Magnitude of the CD4+ and CD8+ T‐cell response to RV

**Figure 2:**
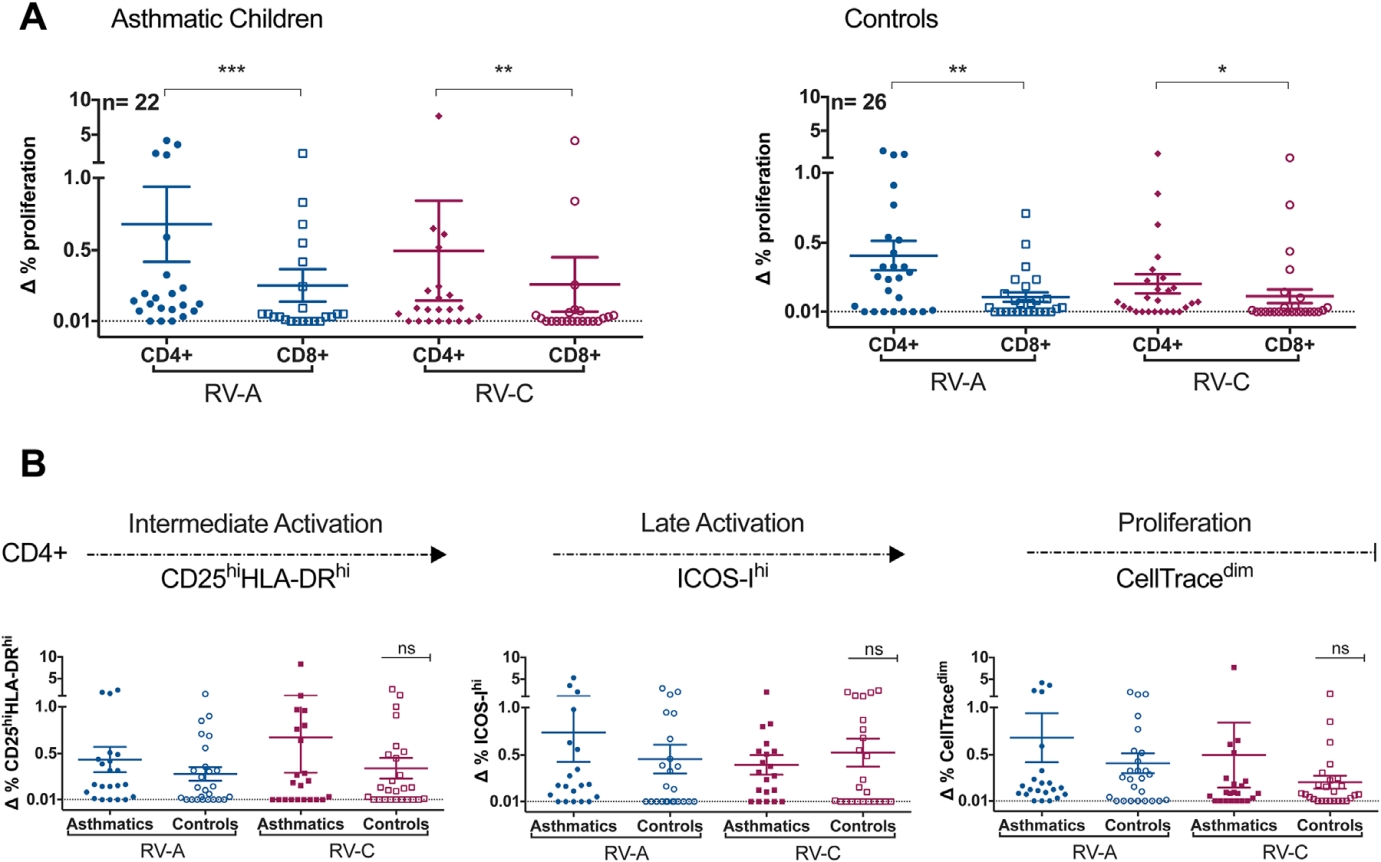
(A) Magnitude of CD4+ and CD8+ proliferation in the in vitro response of asthmatic and control children to rhinoviruses epitopes (B) Magnitude of CD4+ T‐cell activation leading to proliferation, showing functional CD4+ T‐cell response in asthmatics and control children to both RV species. ns, *p *> 0.05; *, *p* ≤ 0.05; **, *p* ≤ 0.01; ***, *p* ≤ 0.001; ****, *p* ≤ 0.0001.

**Figure 3:**
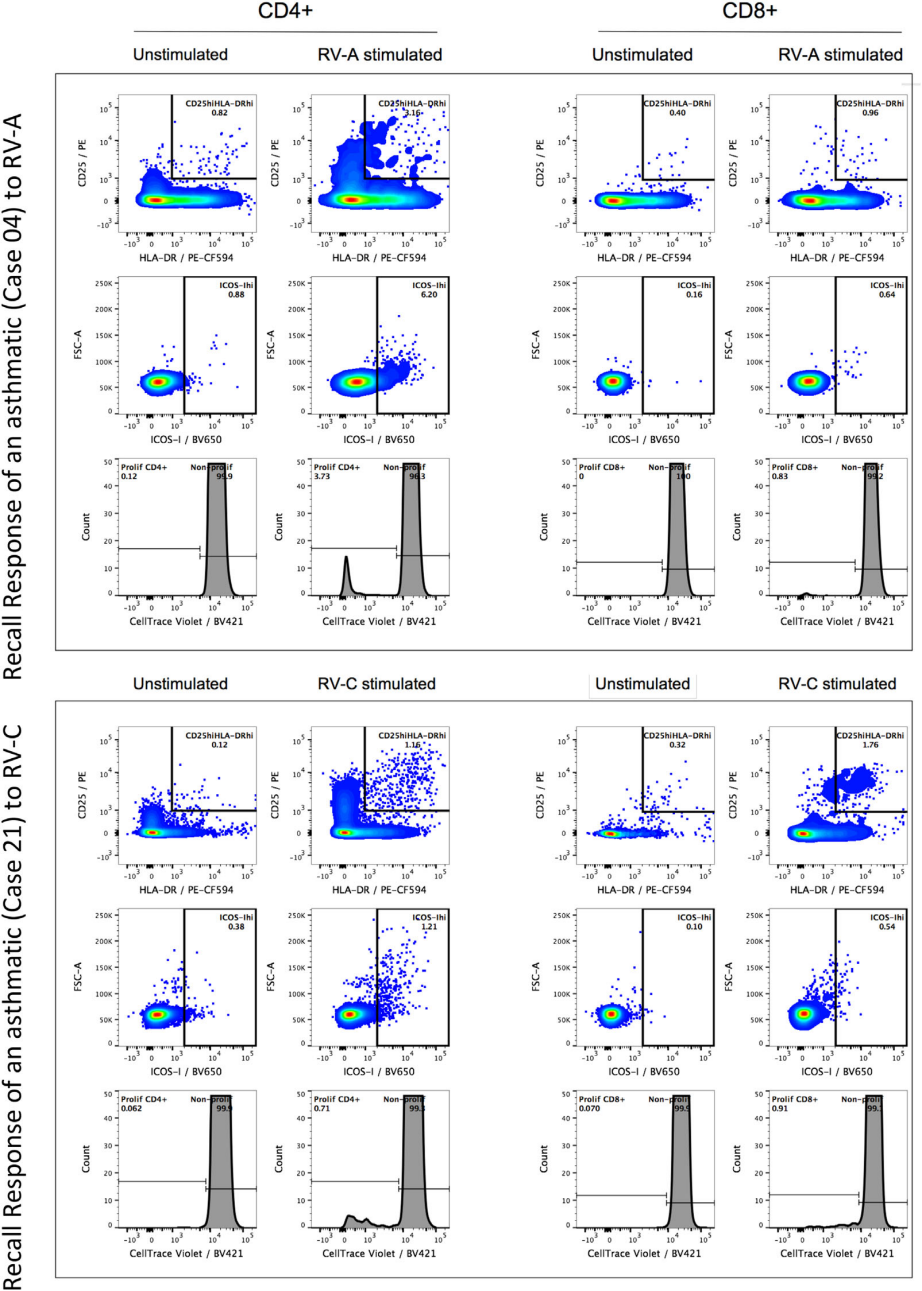
Characteristic T‐cell response to rhinoviruses species A and C in the in vitro recall response to synthetic peptides of the VP1 capsid protein of RV‐A and RV‐C in two asthmatic children.

**Figure 4:**
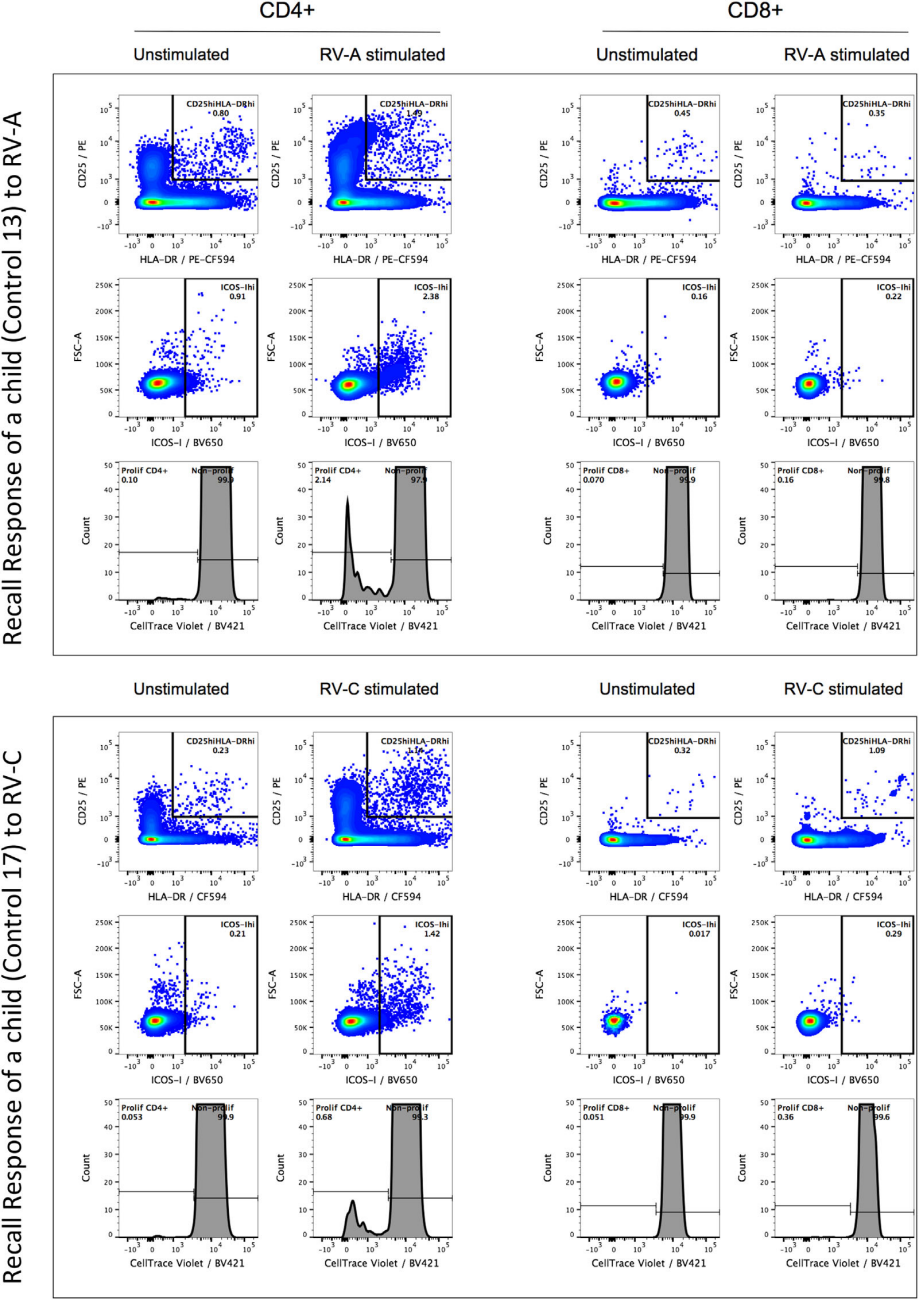
Characteristic T‐cell response to rhinoviruses species A and C in the in vitro recall response to synthetic peptides of the VP1 capsid protein of RV‐A and RV‐C in two healthy control children.

CD4+ T‐cells were the main subset to proliferate (CellTrace^dim^) in response to the RV stimulus in both asthmatic and control groups (Fig. 2A). The magnitude of the memory T‐cell response of asthmatics and controls were compared by analyzing the extent of the expression of activation markers (CD25^hi^HLA‐DR^hi^ and ICOS‐I^hi^) and proliferation (CellTrace^dim^) of CD4+ and CD8+ cells in the response to the RV‐A and RV‐C. There were no significant differences between the magnitude of in vitro CD4+ (Fig. 2B) and CD8+ (not shown) response to rhinoviruses, at both activation and proliferation levels, although the average proliferation of controls was 70% and 55% of that found for the averages of the asthmatic RV‐A and RV‐C responses (Fig. 2B). The characteristic in vitro CD4+ and CD8+ T‐cell recall response to RV‐ A and RV‐C for two asthmatic and two control children is shown in Figures 3 and 4, respectively. The proliferation and activation markers for the controls with (27%) and without positive PCR tests to RV at bleeding did not differ significantly, with the values from PCR positive subjects being found across the whole range of results. These results show that children, independent of their clinical status, have a competent recall of CD4+ memory response to both RV‐A and RV‐C. Tables S1 and S2 summarise the three parameters of CD4+ and CD8+ T cell responses, respectively, against RV‐A and RV‐C in the childhood cohort.

Although of a significantly lower magnitude than the CD4+ T‐cell population, as shown by the activation markers and the frequency of T‐cell proliferation (Fig. 2A), especially in response to RV‐C peptides, the magnitude of the CD8+ proliferative response was significantly higher when compared to unstimulated cultures and was highly correlated with the magnitude of the CD4+ proliferation for both cases (RV‐A, *r* = 0.74, *p *< 0.0001 and RV‐C, *r* = 0.61, *p *< 0.005) and controls (RV‐A, *r* = 0.43, *p *< 0.05 and RV‐C, *r* = 0.55, *p *< 0.005).

### T regulatory cells in the recall T‐cell response to rhinoviruses

Total Tregs (CD4 + CD25^hi^CD127^low^) were calculated as a percentage of the total CD4+ population and were measured in unstimulated PBMCs to compare the frequency of circulating Tregs in asthmatic and control children. The sTregs (CD4 + CellTrace^dim^CD25^hi^CD127^low^) were measured in the CD4+ proliferating fraction of RV‐stimulated cultures to evaluate the ability of asthmatic and control children to develop Tregs under the stimulus of RV.

**Figure 5:**
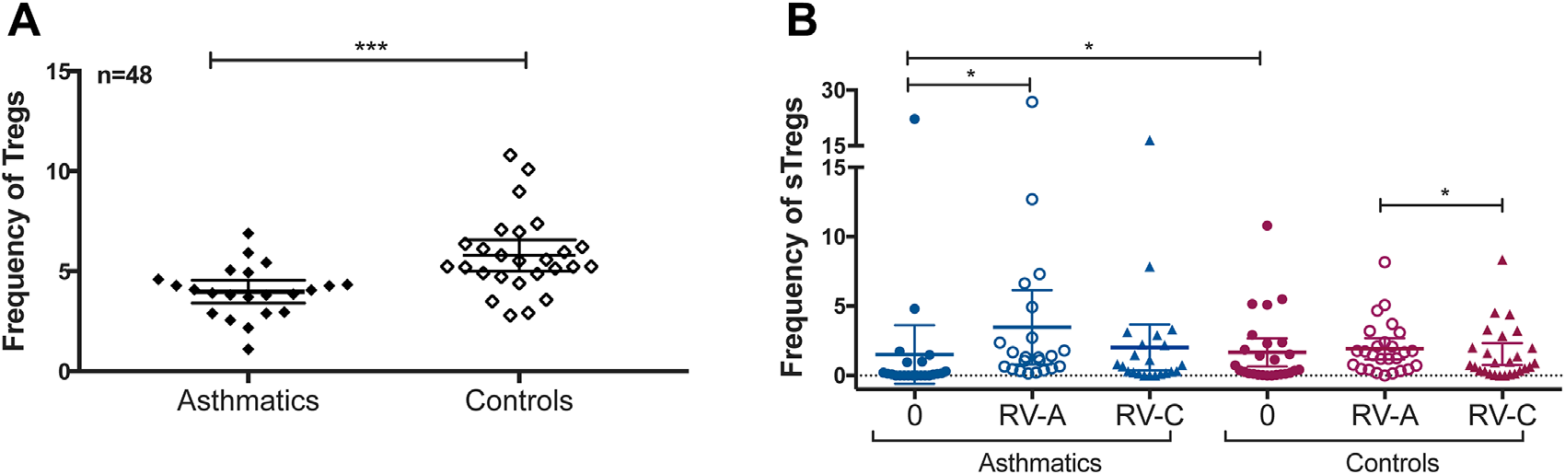
(A) Total Tregs (CD4 + CD25^hi^CD127^low^) calculated as a percentage of the total CD4+ population, measured in unstimulated PBMCs of asthmatic and control children. (B) sTregs (CD4 + CellTrace^dim^CD25^hi^CD127^low^) calculated as a percentage of the proliferating CD4+ subset, measured in background (0), RV‐A and RV‐C stimulated PBMCs of the childhood cohort. ns, *p *> 0.05; *, *p* ≤ 0.05; **, *p* ≤ 0.01; **, *p* ≤ 0.001; ****, *p* ≤ 0.0001.

In agreement with the literature for the frequency of total circulating Tregs in peripheral blood of control children 24, Tregs were found at a frequency of 5.8% ± 1.9 in the control group; the frequency of Tregs in cases was significantly lower (4.0% ± 1.3, *p* ≤ 0.001) (Fig. 5A). Asthmatic children had a higher stimulated Treg response to RV‐A above background (*p* < 0.05), that was not found for the control children, possibly as they already had higher numbers of background sTreg cells compared to asthmatics (*p *< 0.05) (Fig. 3B). The number of sTregs responding to RV‐C was significantly lower than the response to RV‐A for the control group (*p *< 0.02) (Fig. 3B).

## Discussion

This is the first study to compare the T‐cell responses to the different RV species and the peripheral blood responses of healthy and doctor‐diagnosed asthmatic children. A previous study has shown that anti‐RV induced IFN‐γ responses could be detected in cells from broncho‐alveolar lavage of healthy, but not asthmatic subjects upon experimental RV infection, although the data shows that the reduced detection was due to the production of high levels of IFN‐γ without stimulation by the lavage cells from asthmatics but not healthy subjects 25. The study did not find differences in other RV induced cytokines, although the small number of samples analyzed might have precluded a significant estimation of these cytokines. We found indistinguishable recall responses of T‐cells from asthmatics and healthy controls, and for responses to RV‐A and RV‐C peptides. Differences in the magnitude of the RV‐specific T cell memory responses and the activation marker expression are therefore not responsible for the increased anti‐RV‐A IgG in asthmatic children and the anomalously low IgG titres specific to RV‐C found in all subjects 15. The latter indicates that RV‐C does not evade adaptive immune responses per se, but rather that responses may be subject to regulatory events that affect either the specificity of the antibodies or the ability of the response to induce them. The lack of differences in the viral load of healthy and asthmatic adults found after experimental RV‐A infection 25 would be in agreement with the similar T‐cell responses here and suggest that the higher anti‐RV‐A antibody responses might be due to the higher inflammation described by Message et al. 25 or due to more frequent infections. Interestingly, the asthmatics are known to have reduced IL‐15 production 26, a cytokine known to increase antibody responses and induce B‐cell differentiation 27.

CD4+ T‐cell responses are believed to be critical in determining the outcome of many viral infections, including RV 28, 29, 30, 31. In this study, that used peptides selected for the stimulation of CD4+ responses, greater than 70% of all children showing some level of CD4 proliferation. Furthermore, asthmatics’ PBMCs had similar magnitudes of CD4 proliferation as those of control children. In order to evaluate the degree of functionality of this response, three markers of mid to late T‐cell activation were analyzed: ICOS‐I, CD25, and HLA‐DR. ICOS‐I plays a fundamental role in all stages of T‐cell antigen‐depend responses, proving co‐stimulatory signaling at T cell growth, differentiation and effector functions 32, 33, 34. ICOS deficiency results in a reduced memory T cell compartment and defective recall of memory T‐cell response 34. Similarly, HLA‐DR and CD25 are highly expressed on activated memory T cells and participate in effector turnover and persistence of memory T cells 20. Similar levels of CD25, HLA‐DR, and ICOS‐I expression in response to the two RV species were found in both cases and controls and together with the outcomes of CD4+ proliferation, these results suggest that children, independent of their clinical status, have a competent recall of CD4+ memory response to both RV‐A and RV‐C. Additional studies measuring cytokine production by RV‐memory T cells and studies in transcriptome analysis exploring cellular gene‐pathways associated with recall antigenic responses to RVs would complement our findings on functional memory T‐cell response to both RV species. The levels of pro‐inflammatory T‐cell cytokines IFN‐γ, IL‐5, and IL‐13 found in the lung fluids of asthmatics after RV infection 25 were all associated with decreased lung function in acccord with the known effects of these cytokines on lung tissues and in the recruitment of inflammatory cells.

The peptides stimulated much smaller responses from the CD8+ cells. This justified the precaution of measuring CD4+ and CD8+ cells separately but the significance should be viewed recognizing that the assays were optimized for CD4+ responses.

Comprising only 5–10% of the total CD4+ population, Tregs are critically important in maintaining immune homeostasis and preventing exacerbated immune responses. Tregs are fundamental in regulating induction of de novo and memory immune responses to allergens and pathogens, all of which in excess can result in chronic inflammation and damage to epithelial tissue in the airways 35. This would be especially important in the upper and lower respiratory tracts, where a large surface area is constantly exposed to potential antigens. A recent study utilizing class II tetramers to track primary and recall of influenza‐specific memory T‐cell response, showed rapid accumulation of antigen‐specific Tregs in the lungs and associated lymph nodes when compared to the primary infection 36. Furthermore, high CD8+ response and pulmonary inflammation was observed after influenza‐specific memory Treg depletion prior to re‐infection 36 demonstrating the importance of Tregs in controlling immune‐inflammation in response to respiratory viral infections. For rhinoviruses in asthma the Tregs could reduce the Th2 responses associated with adverse lung function and eosinophilic inflammation 25 and reduce inflammatory cytokine production such as IL‐13 25 and produce anti‐inflammatory effects via mediators such as adenosine and TGF‐β that act on inflammatory and endothelial cells. In asthma, a decline in the number of circulating Tregs is associated with increased symptoms of asthma 37, whereas the use of corticosteroids increases the frequency of circulating Tregs in asthmatic children and control symptoms of exacerbation 38. The finding that the RV‐C peptides induced a considerably lower Treg response than the RV‐A peptides not only show a difference in the responses between the two RV species, but is consistent with reports of increased immunopathology produced by RV‐C in lower respiratory tract infections 1, 2. The difference between the Treg responses to RV‐A and RV‐C was however apparently masked in the asthmatics who tended to have much lower overall Treg cell stimulation to RVs.

Our findings indicate that all children, independent of their asthma status, have a competent CD4+ T‐cell recall response to RV‐A and RV‐C. However, we identified significantly lower numbers of circulating Tregs in asthmatic children in comparison to their healthy counterparts and differences of Tregs induced by the antigens of different species. These, and possibly other regulatory immune mechanisms could explain the reduced ability of asthmatics to suppress viral‐induced inflammatory responses in asthma exacerbations.

## Authors' Contribution

CMG and CG contributed to the acquisition and analysis of the experimental data. CMG, AJC, WRT, and BJH contributed to the study design, analysis and interpretation of the data and drafting the article. IL and PNLS contributed to the acquisition of clinical data and interpretation. BJH initiated and led the study. All authors have contributed to the revision of the article.

## CONFLICTS OF INTEREST

The authors declared no conflicts of interest.

## Supporting information

Additional supporting information may be found in the online version of this article at the publisher's web‐site.

**Table S1**. Measurement of CD25^hi^HLA‐DRhi (intermediate activation), ICOS‐Ihi (late activation), and CellTrace^dim^ (proliferation) above background (△ value) in CD4+ of asthmatic and control children in the in vitro recall response to RV‐A and RV‐C epitopes.
**Table S2**. Measurement of CD25^hi^HLA‐DRhi (intermediate activation), ICOS‐I^hi^ (late activation), and CellTrace^dim^ (proliferation) above background (△ value) in CD8+ of asthmatic and control children in the in vitro recall response to RV‐A and RV‐C epitopes.
