# Supplementary material for: T‐cell responses against rhinovirus species A and C in asthmatic and healthy children
Source: Immun Inflamm Dis. 2017 Nov 10;6(1):143–53. doi: 10.1002/iid3.206 (PMC5818445; doi:10.1002/iid3.206)
Supplement: Supplementary file 1 — Table S1. Measurement of CD25hiHLA‐DRhi (intermediate activation), ICOS‐Ihi (late activation), and CellTracedim (proliferation) above background (△ value) in CD4+ of asthmatic and control children in the in vitro recall response to RV‐A and RV‐C epitopes. Table S2. Measurement of CD25hiHLA‐DRhi (intermediate activation), ICOS‐Ihi (late activation), and CellTracedim (proliferation) above background (△ value) in CD8+ of asthmatic and control children in the in vitro recall response to RV‐A and RV‐C epitopes. [file IID3-6-143-s001.docx]

S1 Table. Measurement of CD25^hi^HLA-DR^hi^ (intermediate activation), ICOS-I^hi^ (late activation) and CellTrace^dim^ (proliferation) above background (∆ value) in CD4+ of asthmatic and control children in the *in vitro* recall response to RV-A and RV-C epitopes.

*RV-A non-responder **RV-C non-responder *** RV-A and C non-responder – ICOS-I reading not available

|  |  | **RV-A** | | |  | | **RV-C** | | | | |
| --- | --- | --- | --- | --- | --- | --- | --- | --- | --- | --- | --- |
|  |  | CD25^hi^HLA-DR^hi^ | ICOS-I^hi^ | CellTrace^dim^ | |  | CD25^hi^HLA-DR^hi^ | ICOS-I^hi^ | CellTrace^dim^ |  |  |
|  | **Donor ID** | **Interm Act** | **Late Act** | **Prolif** | |  | **Interm Act** | **Late Act** | **Prolif** |  |  |
| **Asthmatic Children** | Case 01*** | 0.01 | 0.01 | 0.01 | |  | 0.01 | 0.01 | 0.01 |  |  |
|  | Case 02* | 0.01 | 0.01 | 0.01 | |  | 0.64 | 0.42 | 0.01 |  |  |
|  | Case 03 | 1.57 | 2.08 | 2.13 | |  | 0.01 | 0.11 | 0.09 |  |  |
|  | Case 04** | 2.34 | 5.32 | 3.61 | |  | 0.01 | 0.01 | 0.01 |  |  |
|  | Case 05 | 0.04 | 0.01 | 0.01 | |  | 0.27 | 0.54 | 0.09 |  |  |
|  | Case 06 | 0.32 | - | 0.17 | |  | 8.38 | - | 7.69 |  |  |
|  | Case 07 | 0.32 | - | 0.13 | |  | 0.19 | - | 0.01 |  |  |
|  | Case 08 | 0.01 | 0.18 | 0.08 | |  | 0.76 | 2.03 | 0.22 |  |  |
|  | Case 09 | 0.39 | 0.06 | 0.20 | |  | 0.97 | 0.80 | 0.61 |  |  |
|  | Case 10** | 0.49 | 0.56 | 2.35 | |  | 0.01 | 0.01 | 0.01 |  |  |
|  | Case 11 | 0.17 | 0.17 | 0.20 | |  | 0.16 | 0.38 | 0.09 |  |  |
|  | Case 12 | 0.44 | 0.35 | 0.33 | |  | 0.96 | 0.62 | 0.52 |  |  |
|  | Case 13 | 0.42 | 0.12 | 0.12 | |  | 0.80 | 0.54 | 0.25 |  |  |
|  | Case 14** | 0.02 | 0.18 | 0.08 | |  | 0.01 | 0.01 | 0.01 |  |  |
|  | Case 15 | 1.80 | 3.52 | 4.19 | |  | 0.01 | 0.01 | 0.10 |  |  |
|  | Case 16 | 0.15 | 0.19 | 0.10 | |  | 0.21 | 0.13 | 0.10 |  |  |
|  | Case 17 | 0.09 | 0.01 | 0.24 | |  | 0.01 | 0.28 | 0.06 |  |  |
|  | Case 18 | 0.15 | 0.63 | 0.13 | |  | 0.01 | 0.32 | 0.19 |  |  |
|  | Case 19 | 0.01 | 0.01 | 0.15 | |  | 0.04 | 0.13 | 0.04 |  |  |
|  | Case 20 | 0.16 | 0.08 | 0.09 | |  | 1.04 | 0.83 | 0.65 |  |  |
|  | Case 21 | 0.17 | 0.28 | 0.04 | |  | 0.01 | 0.24 | 0.01 |  |  |
|  | Case 22 | 0.51 | 0.98 | 0.59 | |  | 0.29 | 0.51 | 0.17 |  |  |
|  | Control 01 | 0.90 | 0.01 | 0.01 | |  | 2.54 | 2.41 | 0.40 |  |  |
|  | Control 02*** | 0.01 | 0.01 | 0.01 | |  | 0.01 | 0.01 | 0.01 |  |  |
|  | Control 03*** | 0.01 | 0.01 | 0.01 | |  | 0.01 | 0.01 | 0.01 |  |  |
| **Control Children** | Control 04** | 0.71 | 0.95 | 1.45 | |  | 0.01 | 0.01 | 0.01 |  |  |
|  | Control 05 | 0.36 | 0.33 | 0.29 | |  | 0.49 | 0.77 | 0.25 |  |  |
|  | Control 06** | 0.25 | 0.01 | 0.24 | |  | 0.01 | 0.01 | 0.01 |  |  |
|  | Control 07 | 0.14 | - | 0.33 | |  | 0.01 | - | 0.16 |  |  |
|  | Control 08 | 0.04 | - | 0.11 | |  | 0.04 | - | 0.03 |  |  |
|  | Control 09 | 0.01 | 0.14 | 0.01 | |  | 0.14 | 0.01 | 0.18 |  |  |
|  | Control 10** | 0.32 | 0.39 | 0.26 | |  | 0.01 | 0.01 | 0.01 |  |  |
|  | Control 11 | 0.31 | 2.07 | 1.54 | |  | 0.27 | 0.55 | 0.11 |  |  |
|  | Control 12 | 0.01 | 0.67 | 0.33 | |  | 1.00 | 2.03 | 0.85 |  |  |
|  | Control 13 | 0.69 | 1.47 | 2.04 | |  | 0.10 | 0.01 | 0.07 |  |  |
|  | Control 14 | 0.07 | 0.01 | 0.16 | |  | 0.11 | 0.02 | 0.08 |  |  |
|  | Control 15 | 0.01 | 0.01 | 0.02 | |  | 0.21 | 0.49 | 0.17 |  |  |
|  | Control 16 | 0.04 | 0.17 | 0.25 | |  | 1.20 | 1.92 | 1.62 |  |  |
|  | Control 17 | 0.56 | 0.94 | 0.77 | |  | 0.91 | 1.21 | 0.63 |  |  |
|  | Control 18 | 0.15 | 0.01 | 0.54 | |  | 0.58 | 0.87 | 0.31 |  |  |
|  | Control 19*** | 0.01 | 0.01 | 0.01 | |  | 0.01 | 0.01 | 0.01 |  |  |
|  | Control 20 | 0.32 | 0.46 | 0.52 | |  | 0.52 | 0.68 | 0.23 |  |  |
|  | Control 21 | 1.44 | 2.89 | 0.43 | |  | 0.45 | 1.09 | 0.09 |  |  |
|  | Control 22 | 0.85 | 0.01 | 0.91 | |  | 0.01 | 0.01 | 0.05 |  |  |
|  | Control 23 | 0.03 | 0.01 | 0.01 | |  | 0.02 | 0.01 | 0.01 |  |  |
|  | Control 24* | 0.01 | 0.01 | 0.01 | |  | 0.14 | 0.24 | 0.08 |  |  |
|  | Control 25 | 0.11 | 0.38 | 0.33 | |  | 0.13 | 0.16 | 0.04 |  |  |
|  | Control 26 | 0.01 | 0.01 | 0.05 | |  | 0.01 | 0.11 | 0.01 |  |  |
|  |  |  |  |  | |  |  |  |  |  |  |
|  |  |  |  |  | |  |  |  |  | |  |
|  |  |  |  |  | |  |  |  |  | |  |

S2 Table. Measurement of CD25^hi^HLA-DR^hi^ (intermediate activation), ICOS-I^hi^ (late activation) and CellTrace^dim^ (proliferation) above background (∆ value) in CD8+ of asthmatic and control children in the *in vitro* recall response to RV-A and RV-C epitopes.

|  |  | **RV-A** | | |  | | **RV-C** | | | |
| --- | --- | --- | --- | --- | --- | --- | --- | --- | --- | --- |
|  |  | CD25^hi^HLA-DR^hi^ | ICOS-I^hi^ | CellTrace^dim^ | |  | CD25^hi^HLA-DR^hi^ | ICOS-I^hi^ | CellTrace^dim^ |  |
|  | **Donor ID** | **Interm Act** | **Late Act** | **Prolif** | |  | **Interm Act** | **Late Act** | **Prolif** |  |
| **Asthmatic Children** | Case 01*** | 0.01 | 0.01 | 0.01 | |  | 0.01 | 0.01 | 0.01 |  |
|  | Case 02 | 0.07 | 0.01 | 0.01 | |  | 0.44 | 0.01 | 0.01 |  |
|  | Case 03 | 0.31 | 0.56 | 0.42 | |  | 0.15 | 0.13 | 0.05 |  |
|  | Case 04 | 0.56 | 0.48 | 0.83 | |  | 0.01 | 0.01 | 0.05 |  |
|  | Case 05 | 0.01 | 0.01 | 0.02 | |  | 0.10 | 0.06 | 0.08 |  |
|  | Case 06 | 0.10 | - | 0.04 | |  | 3.87 | - | 4.14 |  |
|  | Case 07 | 0.13 | - | 0.01 | |  | 0.02 | - | 0.01 |  |
|  | Case 08 | 0.14 | 0.28 | 0.01 | |  | 0.14 | 0.07 | 0.01 |  |
|  | Case 09 | 0.49 | 0.34 | 0.04 | |  | 0.66 | 0.06 | 0.26 |  |
|  | Case 10 | 0.01 | 0.08 | 2.35 | |  | 0.01 | 0.02 | 0.01 |  |
|  | Case 11 | 0.35 | 0.28 | 0.04 | |  | 0.07 | 0.05 | 0.07 |  |
|  | Case 12 | 0.19 | 0.02 | 0.06 | |  | 0.07 | 0.03 | 0.04 |  |
|  | Case 13 | 0.04 | 0.01 | 0.01 | |  | 0.80 | 0.32 | 0.03 |  |
|  | Case 14* | 0.01 | 0.01 | 0.01 | |  | 0.01 | 0.03 | 0.01 |  |
|  | Case 15 | 0.01 | 0.32 | 0.55 | |  | 0.01 | 0.11 | 0.01 |  |
|  | Case 16 | 0.02 | 0.01 | 0.04 | |  | 0.07 | 0.01 | 0.03 |  |
|  | Case 17** | 0.01 | 0.01 | 0.25 | |  | 0.01 | 0.01 | 0.01 |  |
|  | Case 18** | 0.10 | 0.01 | 0.10 | |  | 0.01 | 0.01 | 0.01 |  |
|  | Case 19 | 0.01 | 0.01 | 0.06 | |  | 0.04 | 0.01 | 0.01 |  |
|  | Case 20 | 0.15 | 0.04 | 0.06 | |  | 1.44 | 0.44 | 0.84 |  |
|  | Case 21** | 0.06 | 0.05 | 0.06 | |  | 0.01 | 0.01 | 0.01 |  |
|  | Case 22 | 0.58 | 0.66 | 0.68 | |  | 0.18 | 0.17 | 0.09 |  |
|  | Control 01 | 0.61 | 0.03 | 0.04 | |  | 2.00 | 1.12 | 0.77 |  |
|  | Control 02*** | 0.01 | 0.01 | 0.01 | |  | 0.01 | 0.01 | 0.01 |  |
|  | Control 03*** | 0.01 | 0.01 | 0.01 | |  | 0.01 | 0.01 | 0.01 |  |
| **Control Children** | Control 04 | 0.36 | 0.42 | 0.71 | |  | 0.03 | 0.01 | 0.01 |  |
|  | Control 05 | 0.41 | 0.46 | 0.49 | |  | 0.49 | 0.23 | 0.15 |  |
|  | Control 06 | 1.31 | 0.25 | 0.07 | |  | 0.01 | 0.01 | 0.04 |  |
|  | Control 07 | 0.14 | - | 0.19 | |  | 0.03 | - | 0.11 |  |
|  | Control 08 | 0.01 | - | 0.09 | |  | 0.48 | - | 0.04 |  |
|  | Control 09 | 0.67 | 0.19 | 0.24 | |  | 0.45 | 0.01 | 0.44 |  |
|  | Control 10** | 0.11 | 0.01 | 0.24 | |  | 0.01 | 0.01 | 0.01 |  |
|  | Control 11 | 0.15 | 0.42 | 0.07 | |  | 0.01 | 0.12 | 0.01 |  |
|  | Control 12 | 0.68 | 0.62 | 0.14 | |  | 0.53 | 0.17 | 0.02 |  |
|  | Control 13** | 0.01 | 0.06 | 0.09 | |  | 0.01 | 0.01 | 0.01 |  |
|  | Control 14 | 0.01 | 0.01 | 0.01 | |  | 0.09 | 0.01 | 0.01 |  |
|  | Control 15 | 0.01 | 0.12 | 0.01 | |  | 0.09 | 0.16 | 0.01 |  |
|  | Control 16 | 0.01 | 0.05 | 0.03 | |  | 0.15 | 0.01 | 1.01 |  |
|  | Control 17 | 0.13 | 0.12 | 0.01 | |  | 0.77 | 0.27 | 0.31 |  |
|  | Control 18 | 0.01 | 0.08 | 0.04 | |  | 0.16 | 0.04 | 0.01 |  |
|  | Control 19* | 0.01 | 0.01 | 0.01 | |  | 0.02 | 0.01 | 0.01 |  |
|  | Control 20* | 0.01 | 0.01 | 0.01 | |  | 0.30 | 0.08 | 0.01 |  |
|  | Control 21 | 0.50 | 0.55 | 0.33 | |  | 0.01 | 0.03 | 0.01 |  |
|  | Control 22 | 0.13 | 0.01 | 0.10 | |  | 0.01 | 0.01 | 0.02 |  |
|  | Control 23*** | 0.01 | 0.01 | 0.01 | |  | 0.01 | 0.01 | 0.01 |  |
|  | Control 24* | 0.01 | 0.01 | 0.01 | |  | 0.11 | 0.01 | 0.05 |  |
|  | Control 25 | 0.02 | 0.01 | 0.01 | |  | 0.12 | 0.02 | 0.01 |  |
|  | Control 26* | 0.01 | 0.01 | 0.01 | |  | 0.01 | 0.10 | 0.01 |  |
|  |  |  |  |  | |  |  |  |  |  |
|  | *RV-A non-responder **RV-C non-responder *** RV-A and C non-responder – ICOS-I reading not available |  |  |  | |  |  |  |  |  |
|  |  |  |  |  | |  |  |  |  |  |
